# Patient communication in Intensive Care Unit-Application in pediatric population

**DOI:** 10.4103/0972-5229.74239

**Published:** 2010

**Authors:** Nagarajan Muthialu

**Affiliations:** Consultant Paediatric Cardiac Surgeon, GKNM Hospital, Coimbatore, Tamilnadu, India

Dear Editor,

The article by Haranath about “Patient Communication (SMS) in Intensive Care Unit (ICU)” is quite interesting to read and gives lots of messages to ponder over.[1] It is really a novel idea that a touch screen or any online keyboard-screen device can be used for gathering information from an intubated but awake patient. But the same may not be applicable for a pediatric population and quite often long-ventilated ones are very small but still awake and have senses in all respects. Music played to their ears and other external communication portals through the parents are quite often reassuring to those children who are awake. The problem accentuates in those who require long-term ventilation with associated problems of cognitive function.

While the issue of communication remains, the aspect of attentiveness needs to be patient-focused. I fully agree with the author that quite often the attention is directed toward a continuous monitor for hemodynamic profile, but one has to remember that there is a patient at the end of the line and patient comes first for any assessment and interaction before any decision is made. This is where all the pictorial charts for easy understanding comes into play and these are very useful even in peripheral units.

Last but not the least, if one has to do a frequent arterial blood sampling for assessment, then there is a definite role for an invasive arterial monitoring which avoids repeated arterial punctures. In adults, placing a radial artery catheter is not a technically challenging one and can be easily maintained for at least a week. Even in children, well-cared peripheral arterial catheters can be maintained for long duration lasting up to 3 weeks without any issues. Someone on ventilation for longer period can also have a central venous catheter in place through easily accessible route (internal jugular vein or subclavian vein or peripherally inserted central catheters depending on personal choice) and can be used for venous gas analysis.
